# Validation and Translation of the 3D-CAM to Turkish in Surgical Intensive Care Patients

**DOI:** 10.4274/TJAR.2025.251888

**Published:** 2025-03-21

**Authors:** Sinem Sarı, Pelin Dilsiz, Tuna Eker, Samet Şahin, Meltem Derya Şahin, Bilge Doğan, Pakize Özçiftçi, Halil Özcan, Ayşenur Dostbil, Mehmet Sinan İyisoy, Oğuz Turan, Fatma Taşkın, Didar Kyenshilik, Meryem Kazaylek, İlker İnce, Alparslan Turan

**Affiliations:** 1 Aydın Adnan Menderes University Faculty of Medicine, Department of Anaesthesiology and Reanimation, Aydın, Türkiye; 2 Outcomes Research Consortium, Houston, Texas, USA; 3 Soma State Hospital, Clinic of Anaesthesiology and Reanimation, Manisa, Türkiye; 4 Soma State Hospital, Clinic of Psychiatry, Manisa, Türkiye; 5 Muğla Sıtkı Koçman University Faculty of Medicine, Department of General Surgery, Muğla, Türkiye; 6 Muğla Sıtkı Koçman University Faculty of Medicine, Department of Psychiatry, Muğla, Türkiye; 7 Aydın Adnan Menderes University Faculty of Medicine, Department of Psychiatry, Aydın, Türkiye; 8 Aydın Adnan Menderes University Faculty of Medicine, Department of Intensive Care Unit, Aydın, Türkiye; 9 Atatürk University Faculty of Medicine, Department of Psychiatry, Erzurum, Türkiye; 10 Atatürk University Faculty of Medicine, Department of Anaesthesiology and Reanimation, Erzurum, Türkiye; 11 Necmettin Erbakan University Faculty of Medicine, Department of Medical Education and Informatics, Konya, Türkiye; 12 Northeast Ohio Medical University, Ohio, United States; 13 Bayburt State Hospital, Clinic of Psychiatry, Bayburt, Türkiye; 14 The Pennsylvania State University, Penn State College of Medicine, Department of Anaesthesiology and Perioperative Medicine, Pennsylvania, United States; 15 Center of Outcome Research, University of Texas, Houston, USA

**Keywords:** 3D-CAM, Delirium, Intensive Care, Turkish Version, Validation

## Abstract

**Objective:**

Delirium is a common condition that can significantly worsen a patient’s clinical status. Timely and accurate detection of this often-overlooked condition is essential for effective prevention and treatment. This study aims to validate the Turkish version of the 3-Minute Diagnostic Interview for Confusion Assessment-defined Delirium (3D-CAM-TR), which has been culturally adapted for surgical intensive care patients.

**Methods:**

This study was conducted in surgical intensive care units and wards at three academic hospitals, including 133 surgical intensive care patients. The 3D-CAM was culturally adapted and translated into Turkish. The 3D-CAM-TR was administered by trained clinicians from the first to the third postoperative day. During this period, delirium diagnosis was made by experienced psychiatrists using the Diagnostic and Statistical Manual of Mental Disorders, Fifth Edition (DSM-5) criteria as the reference standard. All assessors were blinded to each other’s assessment results. The 3D-CAM delirium diagnosis was compared with the reference standard in all patients.

**Results:**

A total of 133 adult patients were assessed over three consecutive days, findings in 399 paired assessments. Compared to the DSM-5-based reference standard, the sensitivity and specificity of the 3D-CAM-TR assessment were found to be 95% and 97%, respectively, for rater 1, and 93% and 99%, respectively, for rater 2, with good inter-rater reliability (Kappa coefficient=0.898, confidence interval=0.84, 0.96).

**Conclusion:**

Our resultings indicate that the 3D-CAM-TR is a dependable and precise instrument for assessing delirium in postoperative intensive care patients.

**Main Points**

• The sensitivity of the Turkish version of the 3-Minute Diagnostic Interview for Confusion Assessment-defined Delirium (3D-CAM-TR) assessment was found to be 95% for rater 1 and 93% for rater 2.

• The specificity of the 3D-CAM-TR assessment were found to be 97% for rater 1, and 99% for rater 2.

• Assessment was have good inter-rater reliability.

## Introduction

Postoperative delirium (PD) is a common complication in geriatric surgical patients. As defined by the Diagnostic and Statistical Manual of Mental Disorders, Fifth Edition (DSM-5), delirium is a transient condition marked by disturbances in attention, awareness, and cognition, with symptoms that develop suddenly and fluctuate over time.^1^ Delirium is associated with accelerated cognitive decline, although there is ongoing debate about whether it serves as a marker or a risk factor for subsequent persistent cognitive impairment.^2, 3^

The exact etiology of PD remains unclear. However, it is recognized as an acute disturbance in cognitive function and/or spatial-temporal perception, which can be diagnosed at the bedside using specific diagnostic tools. PD typically presents with an abrupt onset and a fluctuating course, and without systematic screening, it may be easily overlooked. The condition is characterized by three core features: altered consciousness, changes in cognitive abilities, and a rapid onset.^4^

The DSM-5 is used to establish the definitive diagnosis of delirium. Nevertheless, utilizing it correctly necessitates specialized psychiatric training and education. The most commonly used tool developed for use by non-psychiatric practitioners to help diagnose delirium is the Confusion Assessment Method (CAM). Other tools described include the CAM for intensive care units (CAM-ICU) and the CAM-defined 3 Minute Diagnostic Interview for Delirium (3D-CAM).^1^

The 3D-CAM can be completed in an average of three minutes and has excellent diagnostic test properties with 95% sensitivity and 94% specificity compared to a reference standard based on a comprehensive clinical evaluation.^5^ The 3D-CAM is a concise interview that utilizes verbal responses complete the CAM diagnostic algorithm.^6^ By providing a short, repeatable method for detecting delirium, the 3D-CAM facilitates case finding among hospitalized frail elderly patients.

Although the 3D-CAM has been translated and validated into many languages, there is currently no Turkish translation or validation available. The aim of our study is to translate the 3D-CAM into Turkish and validate the Turkish version 3D-CAM (3D-CAM-TR) in surgical patients.

## Methods

This study was conducted prospectively to translate and validate the reliability of the Turkish version of the 3D-CAM in surgical intensive care patients. The research protocol received approval from the Aydın Adnan Menderes University Faculty of Medicine, Non-Interventional Clinical Research Ethics Committee (date: 28.01.2021, approval no.: 2021/28). The study was registered with Clinical Trials under the number NCT04853706. The multicenter study was carried out. All enrolled patients or their proxies provided written informed consent.

### Translation and Back Translation

The forward translation process, which involves translating the original version into the target language, was carried out independently by two bilingual experts: a specialist doctor of Turkish descent who has lived in the United States for an extended period (AT), and a medical student, also of Turkish descent, who was born and raised in the United States (OT). Both translators independently translated all items of the 3D-CAM, including response options and instructions, into Turkish. The initial translation was then reviewed by Dr. Edward R. Marcantonio, the original developer and validator of the 3D-CAM. After incorporating the revisions he suggested, the translation received his formal approval.

**Figure 1:**
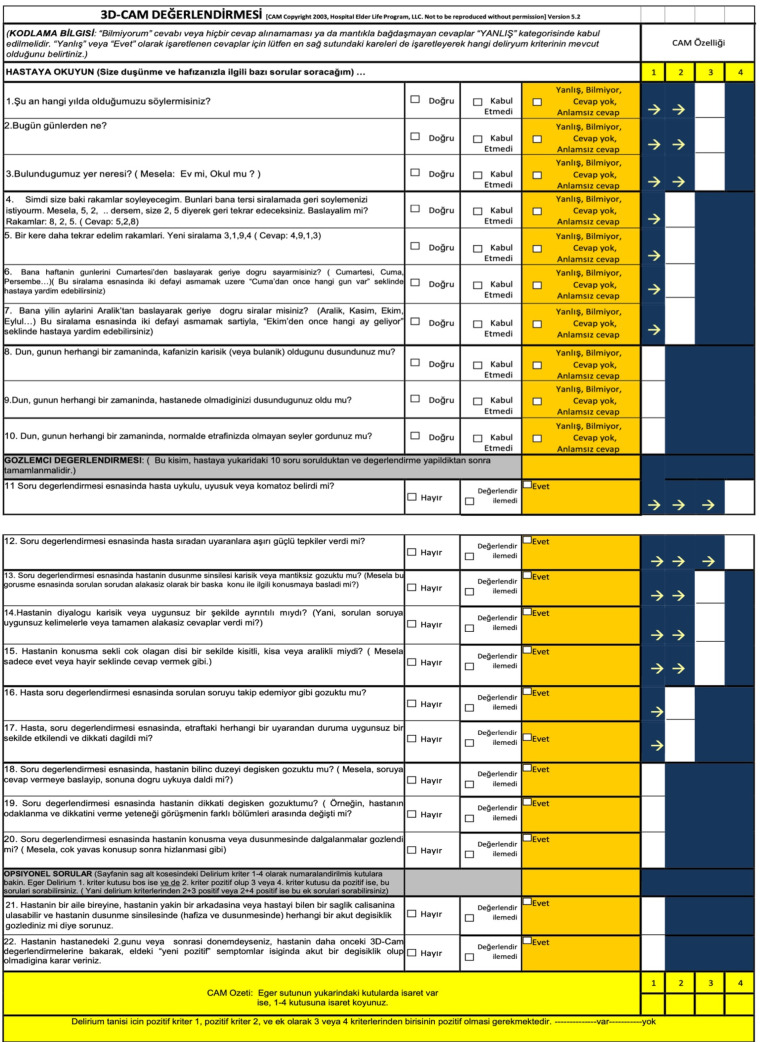
Turkish Version of 3D-CAM.

To identify potential conceptual inaccuracies, a back-translation process was employed, whereby the translated text was retranslated from the target language back into the source language. Any back-translations that deviated from the intended meaning were revised in the Turkish version, back-translated again, and subjected to further review. This iterative process continued until the principal developer gave final approval for the reverse translation (Figure 1).

### Participants

To be eligible for participation, individuals met the following inclusion criteria: (a) be at least 18 years of age and (b) have an American Society of Anesthesiologists (ASA) classification. Physical status 1-3. (c) admitted to the postoperative critical care unit and are expected to stay in the hospital for more than 48 hours. Additionally, patients with an Mini-Mental State Examination (MMSE) score of 20 or higher are included, while those with dementia are excluded.

Exclusion criteria: (a) Patients who declined to participate, (b) Patients with significant visual or auditory impairment/disability or the presence of endotracheal intubation that may impede communication, (c) The presence of a significant psychiatric condition, such as bipolar disorder, major depression, schizophrenia, Alzheimer’s disease, dementia, or parkinsonism, and (d) profound sedation or unconsciousness. ASA physical status IV or V refers to patients who have severe systemic disease posing a continuous threat to life (IV) or patients who are not expected to survive without the surgery (V). (e) Patients receiving surgical procedures with a duration of less than one hour.

### Enrollment and Baseline Data Collection

The assessment of eligibility and obtaining patient consent were conducted during the preoperative consultation. Demographic and historical medical information, including medication use, was obtained during this appointment.

The MMSE was performed. Patients with an MMSE score above 20 were included in the study. Patients excluded for any reason, including technical issues or contraindications, were recorded.

### Delirium Assessment

### 3D-CAM Delirium Assessment 

Before the study period, researchers from all centers participated in web-based instruction on the 3D-CAM. The assessment of delirium was conducted by two different clinicians utilizing the 3D-CAM tool each evening from 18:00 to 20:00 for a period of three days postoperatively. If the patient was transferred from the ICU to the ward, the assessment was conducted in the ward.

### DSM-5 Delirium Assessment

An impartial psychiatrist researcher, who was blind to the 3D-CAM assessment results, assessed the patients based on DSM-5 criteria within a 3-minute timeframe following the 3D-CAM evaluation.

### Statistical Analysis

### Sample Calculation

A prior study found the incidence of delirium to be approximately 13%, with a sensitivity of 85% and specificity of 97%. We set the confidence interval (CI) width at 0.2 and determined that 377 assessments were needed.^1^

### Outcome Analysis

Mean and standard deviation were provided for numerical variables, and frequency and percentage statistics were provided for categorical variables. Cohen’s kappa statistic was used to calculate inter-rater agreement, with a 95% CI provided (Additionally, McNemar test results were included). Analyses were performed using R 4.3.2 (R Core Team, 2024). A *P* value of <0.05 was considered significant.

## Results

**Figure 2:**
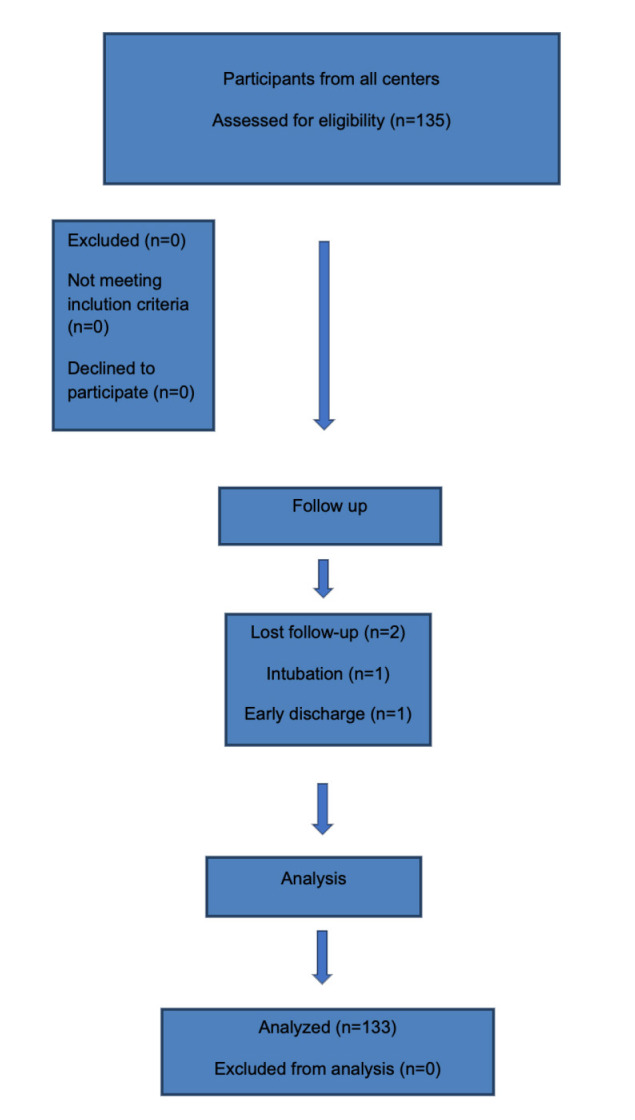
Flow chart.

Between October 2021 and December 2024, 135 patients from this group who met the inclusion criteria and provided written informed consent were included in the study; 2 of these patients did not complete the study, and the analysis was completed with 133 patients (Figure 2). The mean age of the enrolled patients was 60.63±15.55 years, and the MMSE score was 27.8±2.60. Socio-demographic and perioperative data are presented in Table 1.

A total of 399 paired assessments were conducted over a period of three consecutive days for each patient. Based on the psychiatrist’s evaluation using the DSM-5, 19.55% (26 out of 133) of the patients encountered at least one episode of delirium.

When compared to the reference standard DSM-5 psychiatrist evaluation, the sensitivity and specificity of the 3D-CAM-TR assessment were 95% and 97% for rater 1, and 93% and 99% for rater 2, respectively (Table 2). The inter-rater reliability, expressed as the Kappa coefficient, was found to be 0.898 with a CI of 0.84 to 0.96.

A total of 274 paired assessments were conducted in the ICU, while 125 assessments were conducted in the ward, encompassing all enrolled patients. The sensitivity of the 3D-CAM-TR in the ICU was 92% for rater 1 and 94% for rater 2; the specificity was 96% for rater 1 and 99% for rater 2 (Table 3).

The sensitivity of the 3D-CAM-TR in the ward was 100% for both rater 1 and rater 2; the specificity was 98% for rater 1 and 100% for rater 2 (Table 4).

## Discussion

This study provides evidence that the Turkish version of 3D-CAM is a dependable instrument for evaluating delirium in patients, with an MMSE score of 20 and above, who are receiving intensive care after surgery. When compared to the reference standard DSM-5 psychiatrist evaluation, the sensitivity and specificity of the 3D-CAM-TR assessment were 95% and 97% for rater 1, and 93% and 99% for rater 2, respectively. 3D-CAM-TR also yielded positive results when evaluated separately for patients in the ICU and the ward. A total of 274 paired assessments were conducted in the ICU, while 125 assessments were conducted in the ward, encompassing all enrolled patients. The sensitivity of the 3D-CAM-TR in the ICU was 92% for rater 1 and 94% for rater 2; the specificity was 96% for rater 1 and 99% for rater 2.

Delirium is frequently overlooked in clinical settings, and more than 28 diagnostic techniques have been created and implemented to aid in its screening.^7, 8^ These tools have greatly enhanced the efficiency and precision of diagnosing delirium. Among these tools, CAM has been proposed as the most effective.^7, 8^ Nevertheless, one disadvantage of CAM is that, despite extensive training, there may still be inconsistencies in the assessment criteria used by different assessors.^7^ Hence, the proficiency of the evaluator’s interrogative abilities might greatly influence the outcomes of the evaluation. The 3D-CAM, a derivative of CAM, offers a concise and organized assessment technique to expedite and streamline the diagnostic procedure.^5^ The 3D-CAM’s capacity to conduct evaluations within just three minutes is a notable benefit for clinical practice.

This study confirmed the efficacy and dependability of the 3D-CAM-TR in both the ICU (without the use of endotracheal intubation) and ward settings for surgical patients. Several diagnostic methods, such as CAM-ICU and the critical Care Delirium Screening Checklist, have been utilized to diagnose delirium in patients in ICUs.^9^ Nevertheless, the comparison between these tools and the 3D-CAM has only been conducted in a limited number of studies. A study was conducted with 101 elderly patients (aged 75 years or older) who were not in the ICU. The study found that the 3D-CAM method was more effective than the CAM-ICU method in identifying delirium.^10^

In a study similar to our study validating the 3D-CAM-CN, it was reported to be a reliable tool for diagnosing delirium in postoperative patients.^1^ They highlighted the strengths of the study, including comprehensive pre-study preparation and strict criteria provided by a panel of psychiatrists. In our study, we planned to demonstrate the 3D-CAM application through online training. Subsequently, evaluators applied the 3D-CAM-TR without supervision. We believe that extended training periods reduce the efficiency of test administration, which is one of the test’s advantages. We suggest that future research could explore evaluations after different levels of training and determine the optimal training duration.

### Study Limitations

Our study’s strengths include a culturally appropriate translation and a sufficient sample size. However, there are also limitations. Types of delirium identified in the study were not recorded, so the reliability of the test among hypoactive, hyperactive, and mixed types could not be evaluated. The reliability of the test in patients with major cognitive impairment has been previously established. However, since we included only patients with an MMSE score above 20 and no diagnosis of cognitive impairment, the reliability in the group with major cognitive impairment could not be assessed.

## Conclusion

This study successfully linguistically validated the 3D-CAM for use in the Turkish population, enabling its application for assessing delirium in Turkish-speaking patients. The Turkish version of the questionnaire is now ready for use in post-surgical patients who are not intubated and who do not have cognitive impairment.

## Ethics

**Ethics Committee Approval:** The research protocol received approval from the Aydın Adnan Menderes University Faculty of Medicine, Non-Interventional Clinical Research Ethics Committee (date: 28.01.2021, approval no.: 2021/28).

**Informed Consent:** All enrolled patients or their proxies provided written informed consent.

## Figures and Tables

**Table 1. Socio-demographic and Perioperative Data:** 

**Variable**	**n = 1331**
**Age, years**	60.63±15.55
**Gender**	
Female	44.00 (33.08%)
Male	89.00 (66.92%)
**Height, cm**	168.92±9.10
**Body weight, kg**	68.49±13.37
**Education level**	
Secondary	87.00 (65.41%)
High school	32.00 (24.06%)
University	9.00 (6.77%)
Postgraduate	5.00 (3.76%)
**Marital status**	
Married	111.00 (83.46%)
Never married	10.00 (7.52%)
Divorced	3.00 (2.26%)
Widowed	9.00 (6.77%)
**Illnesses**	
Stroke	3 (1.4%)
Hypertension	57 (27.3%)
CAD	32 (15.3%)
Arrhythmia	7 (3.3%)
COPD	15 (7.2%)
DM	28 (13.4%)
Hyperlipidemia	5 (2.4%)
CRD	10 (4.8%)
Hyperthyroidism	1 (0.5%)
Hypothyroidism	1 (0.5%)
None	50 (23.9%)
**ASA**	
1	15.00 (11.28%)
2	75.00 (56.39%)
3	43.00 (32.33%)
**MMSE, score**	27.80±2.60

Data was presented as mean (standard deviation) or number (percentage)

CAD, coroner artery disease; COPD, chronic obstructive pulmonary disease; DM, diabetes mellitus; CRD, chronic renal disease; ASA, American Society of Anesthesiologists; MMSE, mini-mental state examination

**Table 2. Raters’ Concordance with the Reference Standard:** 

-	**Reference Standard by DSM-5**		-	-
-	**Positive, n = 61^1^**	**Negative, n = 338^1^**	**Kappa**	**p^2^**
**Rater 1**	-	-	0.871	0.061
Positive	58 (95.1%)	11 (3.3%)	-	-
Negative	3 (4.9%)	327 (96.7%)	-	-
**Rater 2**	-	-	0.932	>0.9
Positive	57 (93.4%)	3 (0.9%)	-	-
Negative	4 (6.6%)	335 (99.1%)	-	-

^1^n (%)

^2^McNemar’s chi-squared test with continuity correction

**Table 3. Raters’ Concordance with the Reference Standard in the Intensive Care Unit:** 

-	**Reference Standard by DSM-5 in ICU**		**-**	
-	**Positive, n = 50^1^**	**Negative, n = 224^1^**	**Kappa**	**p^2^**
**Rater 1**	-	-	0.860 (0.78, 0.94)	0.15
Positive	47 (94.0%)	9 (4.0%)	-	-
Negative	3 (6.0%)	215 (96.0%)	-	-
**Rater 2**	-	-	0.914 (0.85, 0.98)	>0.9
Positive	46 (92.0%)	3 (1.3%)	-	-
Negative	4 (8.0%)	221 (98.7%)	-	-

^1^n (%)

^2^McNemar’s chi-squared test with continuity correction

ICU, intensive care unit.

**Table 4. Raters’ Concordance with the Reference Standard in the Ward:** 

-	**Reference Standard by DSM-5 in ward**		**-**	
-	**Positive, n = 11^1^**	**Negative, n = 114^1^**	**Kappa**	**p^2^**
**Rater 1**	-	-	0.908 (0.78, 1)	0.5
Positive	11 (100.0%)	2 (1.8%)	-	-
Negative	0 (0.0%)	112 (98.2%)	-	-
**Rater 2**	-	-	1.00 (1.1)	-
Positive	11 (100.0%)	0 (0.0%)	-	-
Negative	0 (0.0%)	114 (100.0%)	-	-

^1^n (%)

^2^McNemar’s chi-squared test with continuity correction.
